# Exogenous transforming growth factor‐**β**1 enhances smooth muscle differentiation in embryonic mouse jejunal explants

**DOI:** 10.1002/term.2409

**Published:** 2017-04-27

**Authors:** Riccardo Coletta, Neil A. Roberts, Michael J. Randles, Antonino Morabito, Adrian S. Woolf

**Affiliations:** ^1^ Institute of Human Development, Faculty of Medical and Human Sciences University of Manchester UK; ^2^ Paediatric Autologous Bowel Reconstruction and Rehabilitation Unit, Department of Paediatric Surgery, Royal Manchester Children's Hospital Central Manchester Foundation Trust Manchester UK; ^3^ Wellcome Trust Centre for Cell‐Matrix Research, Faculty of Life Sciences University of Manchester Manchester UK; ^4^ Institute of Inflammation and Repair, Faculty of Medical and Human Sciences University of Manchester Manchester UK; ^5^ Department of Paediatric Nephrology, Royal Manchester Children's Hospital Central Manchester Foundation Trust Manchester UK

**Keywords:** culture, intestine, longitudinal, mesenchyme, microarray, organ

## Abstract

An *ex vivo* experimental strategy that replicates *in vivo* intestinal development would in theory provide an accessible setting with which to study normal and dysmorphic gut biology. The current authors recently described a system in which mouse embryonic jejunal segments were explanted onto semipermeable platforms and fed with chemically defined serum‐free media. Over 3 days in organ culture, explants formed villi and they began to undergo spontaneous peristalsis. As defined in the current study, the wall of the explanted gut failed to form a robust longitudinal smooth muscle (SM) layer as it would do *in vivo* over the same time period. Given the role of transforming growth factor β1 (TGFβ1) in SM differentiation in other organs, it was hypothesized that exogenous TGFβ1 would enhance SM differentiation in these explants. *In vivo*, TGFβ receptors I and II were both detected in embryonic longitudinal jejunal SM cells and, in organ culture, exogenous TGFβ1 induced robust differentiation of longitudinal SM. Microarray profiling showed that TGFβ1 increased SM specific transcripts in a dose dependent manner. TGFβ1 proteins were detected in amniotic fluid at a time when the intestine was physiologically herniated. By analogy with the requirement for exogenous TGFβ1 for SM differentiation in organ culture, the TGFβ1 protein that was demonstrated to be present in the amniotic fluid may enhance intestinal development when it is physiologically herniated in early gestation. Future studies of embryonic intestinal cultures should include TGFβ1 in the defined media to produce a more faithful model of *in vivo* muscle differentiation. Copyright © 2017 The Authors Journal of Tissue Engineering and Regenerative Medicine Published by John Wiley & Sons, Ltd

## 1 Introduction

The mature intestine propels, digests and absorbs food and nutrients. It is essential for health, as evidenced by the morbidity and mortality associated with short bowel syndrome (Coletta et al., 2014; Wales and Christison‐Lagay, 2010) and necrotizing enterocolitis (Hall et al., 2013). Moreover, defining how the intestine develops is of fundamental biological importance and such knowledge may help understand the mechanisms of gut malformations (Masumoto et al., 1999) and regeneration after injury (Maghsoudlou et al., 2014; Miyoshi et al., 2012).

In mice, the gut tube forms at embryonic day 9 (E9) when endoderm becomes surrounded by splanchnic mesoderm. Mesodermal cells then form a multilayered mesenchyme around the epithelial tube, and the gut begins to pattern into the oesophagus, stomach and intestines (Cervantes et al., 2009; Wells and Spence 2014; Wilm et al., 2005). Between mouse E14 and E17, anatomically equivalent to 7 and 9 weeks in human gestation (Sadler, 1990), key events in differentiation and morphogenesis occur in the small intestine (Coletta et al., 2016). These include the formation of villi, each comprising an epithelium with a mesenchymal core, which protrude into the lumen. In the same time frame, a longitudinal smooth muscle (SM) layer forms around an already present circular SM layer.

An *ex vivo* experimental strategy that replicates *in vivo* intestinal development would in theory provide an accessible setting with which to study normal and dysmorphic gut biology. A system was recently described in which mouse E14 jejunal segments were explanted onto semipermeable platforms and fed with chemically defined serum‐free media (Coletta et al., 2016). Over 3 days in organ culture, explants formed villi and they began to undergo spontaneous peristalsis (Coletta et al., 2016). Nevertheless, as defined in the current study, the wall of the explanted gut fails to form a robust longitudinal SM layer and so does not completely match its *in vivo* counterpart.

Previous studies have implicated transforming growth factor β1 (TGFβ1) in postnatal gut maturation and in regeneration following injury. In the mature intestine, TGFβ1 is detected in the tips of villi (Barnard et al., 1993) and exogenous TGFβ1 inhibits the proliferation of intestinal epithelia in cell culture (Yamada et al., 2013). After colonic injury, TGFβ is implicated in establishing new crypts containing quiescent epithelia (Miyoshi et al., 2012). By contrast, the possible roles of TGFβ1 in embryonic gut development have been little studied. Notably, however, intestines of mice lacking fibroblast growth factor 9 (FGF9) display premature myogenesis and upregulated TGFβ signalling (Geske et al., 2008). Furthermore, TGFβ1 has also been implicated in supporting SM differentiation in diverse other tissues including urinary bladder mesenchyme (Liu et al., 2010), amniotic stem cells (Ghionzoli et al., 2013) and neural crest cells (Huang et al., 2011).

Given the above observations, it was here hypothesized that exogenous TGFβ1 would enhance SM differentiation in embryonic mouse jejunal explants. The growth and molecular composition of explants were characterized and quantified using whole mount imaging, immunohistochemistry and ribonucleic acid (RNA) microarrays. These techniques revealed that exogenous TGFβ1 led to the differentiation of longitudinal SM in explants. At the level of the transcriptome, TGFβ1 had effects correlating with the observed tissue changes. Moreover, it is shown that, *in vivo*, the physiologically herniated intestine is in close proximity to amniotic fluid, a source of TGFβ1 protein.

## 2 Materials and methods

### 2.1 Organ culture and amniotic fluid analyses

Reagents were obtained from Sigma–Aldrich, UK, unless otherwise stated. Animal experiments were approved by the Institutional Review Board of the Registered Medical and Scientific Departments of the University of Manchester. Wild type (CD1) mice were mated overnight in the University's Biological Services Facility, with the morning of the vaginal plug designated embryonic day 0 (E0). After Schedule 1 killing, amniotic fluid was collected from E14 and E17 embryos via a 16‐gauge needle. Fluid was centrifuged and enzyme‐linked immunosorbent assays used to quantify TGFβ1 (ab119557, Abcam, Cambridge, UK) in supernatants. The embryonic jejunum was dissected and a 2–3 mm section from each animal was used for histology or organ culture. For the latter, as described (Coletta et al., 2016), each E14 rudiment was placed onto semipermeable 0.4‐μm pore polytetrafluoroethylene culture plate inserts (Millicell; Millipore, UK) and maintained at 37 °C in an atmosphere of air/5% CO_2_. Each culture was fed with 1 mL of defined serum‐free media, placed underneath and touching the platform. Basal media comprised Dulbecco's modified Eagle's medium/F12 (Gibco BRL, Paisley, UK), insulin (10 mg/L), sodium selenite (5 mg/L), and transferrin (5.5 mg/L). Enzyme‐linked immunosorbent assay was used to quantify TGFβ1 in conditioned media of organs fed basal media. In some experiments, this was supplemented with recombinant human TGFβ1 (R&D Systems, Abingdon, UK) at 5 or 50 ng/mL, concentrations used in organ culture of embryonic mouse kidneys (Clark et al., 2001; Rogers et al., 1993) and salivary glands (Hardman et al., 1994). Explants were maintained for 3 days, previous observations having shown that longer cultures were less viable (Coletta et al., 2016). In some experiments, a polyamide 10/0 suture (Ethicon, New Jersey, USA) was threaded through the rudiment lumen to keep the explant straight and ensure generation of precise transverse histology sections.

### 2.2 Histology

Tissues were fixed for 30 min in 4% paraformaldehyde, rinsed in 1% phosphate‐buffered saline (PBS), dehydrated in ice cold methanol and stored in –20 °C. After rehydration, samples were embedded in 25% fish gelatin and cryosectioned at 10 μm. Transverse sections were fixed in acetone for 5 min, washed with ice cold 1% PBS and permeabilized in 1% PBS, 0.1% Triton X‐100 for 10 min. Mouse on Mouse Blocking Reagent (MOM™, Vector Laboratories, Peterborough, UK) was used to block endogenous immunoglobulins. Sections were incubated for 30 min with primary antibodies against: α‐smooth muscle actin (αSMA; A2547, Santa Cruz Biotechnology, Wembley, UK: 1:200), a visceral muscle cytoskeletal protein; E‐cadherin (ab76055, Abcam; 1:200), an epithelial cell‐cell adhesion protein; Ki67 (ab16667, Abcam; 1:100), a nuclear proliferation associated protein; TGFβ receptor I (TGFβRI; ab31013, Abcam; 1:200); and TGFβRII (ab186838, Abcam; 1:200). After washing, fluorescent secondary antibodies were applied for 1 h. Sections were counterstained with 4′, 6‐diamidino‐2‐phenylindole (DAPI) to detect nuclei. Microscopy was performed as described (Coletta et al., 2016) and images were processed and analysed using ImageJ (http://rsb.info.nih.gov/ij) and Adobe Photoshop CS6. In a typical organ culture experiment, the jejunal rudiments in 0, 5 and 50 ng/mL TGFβ1 originated from three embryo littermates and results were paired for statistical analyses. For individual parameters, the value for each rudiment was the average of measurements made in three sections approximately 50 μm apart. Datasets were subjected to the Shapiro–Wilk test to determine whether or not they were compatible with a normal distribution; thereafter, parametric (two‐tailed *t* test) or nonparametric (two‐tailed Wilcoxon) analyses were applied, as appropriate. Fisher's exact test (two‐tailed) was used to compare proportions of organs containing a longitudinal SM layer.

### 2.3 RNA microarrays

RNA was isolated using the RNeasy Plus kit (Qiagen, Manchester, UK). RNA quality was assessed, and concentrations measured, using a NanoDrop 2000 spectrophotometer. cDNA was generated using a High Capacity RNA‐to‐cDNA Kit (Applied Biosystem, Warrington, UK). For whole transcriptome microarray expression analyses, amplified sense‐strand cDNA (Ambion WT Expression Kit®, Ambion, UK) was generated from 100 ng of total RNA. Fragmentation and labelling (Affymetrix Genechip WT Terminal labelling kit®, Affymetrix, High Wycombe, UK) and subsequent hybridization using Affymetrix Genechip Mouse Exon 1.0 ST Array® was performed at the Genomic Technologies Core Facility at the University of Manchester. Data (ArrayExpress database http://www.ebi.ac.uk/arrayexpress accession number E‐MTAB‐4509) were processed and analysed using Partek Genomics Solution (version 6.5, © 2009, Partek Inc) with these options: probe sets of the core subset were quantile normalised and robust multiarray background correction applied. Exons were summarized to genes by calculating the mean of the exons (log 2). Validation and gene enrichment strategies consisted of the following steps. To establish relationships and compare variability between replicate arrays and experimental conditions, principal components analysis was used. Principal components analysis was chosen for its ability to reduce the effective dimensionality of complex gene‐expression space without significant loss of information (Quackenbush, 2001). Next, differential expression in response to treatment was calculated using Cyber‐T (Baldi and Long, 2011). Correction for false discovery rates was done using the method of QVALUE (Storey and Tibshirani, 2003). To validate array results, quantitative polymerase chain reaction (QPCR) TaqMan Gene Expression assays were undertaken for selected transcripts altered by TGFβ1: upregulated *Krt17* (encoding keratin 17, an epithelial cytoskeletal molecule; Mn00495207_m1), *Ctsw* (cathepsin W, a cysteine proteinase; Mn00515599_m1), *Anpep* (aminopeptidase N, a small intestinal peptidase; Mn00476227_m1), *Col8a1* (collagen VIIIα1, an extracellular matrix molecule; Mn01344185_m1), *Lgr5* (leucine‐rich repeat containing G protein‐coupled receptor 5, expressed in intestinal stem cells; Mn00495207_m1), *Eln* (elastin, an extracellular matrix molecule; Mn00514670_m1), *Angpt1* (angiopoietin 1, a vascular growth factor; Mn00456503_m1), *Sfrp1* (secreted frizzled‐related protein 1, a Wingless‐type MMTV integration site family (WNT) signalling pathway regulator; Mn00489161_m1); and downregulated *Fxyd2* (FXYD domain containing ion transport regulator 2, a regulatory subunit of Na,K‐ATPase; Mn00446358_m1), *Gmp6a* (glycoprotein M6A, a neuronal glycoprotein; Mn00463812_m1); *Mlxipl* (MLX interacting protein‐like, an enteroendocrine transcription factor; Mn02342723_m1); *Pten* (phosphatase and tensin homologue, a tumour suppressor; Mn00477208_m1); and *Sfrp2* (a WNT signalling pathway regulator; Mn01213947_m1). QPCR was also undertaken for *Tgfβ1* (Mn01178820_m1) and the housekeeping transcript *Gapdh* (glyceraldehyde 3 phosphate dehydrogenase; Mn99999915_m1). The assay plate was placed in the StepOne^TM^ Real Time PCR‐system (Applied Biosystems). Samples were heated to 50 °C for 2 min followed by 95 °C for 10 min. Cycling conditions were: 95 °C for 15 s followed by 60 °C for 1 min for 40 cycles.

### 2.4 Clustering and gene ontology enrichment analysis

Z‐transformed mean normalised intensities were used for hierarchical clustering of microarray data. Agglomerative hierarchical clustering was performed using MultiExperiment Viewer (version 4.8.1) (Saeed et al*.* 2003). Normalised probeset intensities were hierarchically clustered on the basis of Euclidean distance, and distances between probesets were computed using a complete‐linkage matrix. Clustering results were visualized using MultiExperiment Viewer (version 4.8.1). Gene clusters identified by hierarchical clustering were analysed using DAVID gene ontology enrichment analysis (Huang da et al., 2009a, b). Keywords with fold enrichment ≥1.5, Bonferroni‐corrected *p* value <0.05, EASE score (modified Fisher's exact test) <0.05 and at least two transcripts per keyword were considered significantly over‐represented. These data were imported into Cytoscape (version 2.8.1) (Shannon et al., 2003) and analysed using the Enrichment Map plugin (Merico et al., 2010). The following criteria were used to generate the networks: false discovery rate (Benjamini–Hochberg) cut‐off <0.01, and similarity cut‐off >0.6. The networks generated were subjected to a Markov Cluster Algorithm to generate distinct sub networks. Transcripts with significantly altered expression in the microarray were searched against Jackson laboratory MGI‐Mouse gene expression database to identify *muscle tissue* (TS12‐28) markers. To identify SM‐specific transcripts, the following data query was used: *find genes where expression is detected in SM tissue (TS20‐28) and expression is not detected or analysed in skeletal muscle (TS20‐28)*.

## 3 Results

### 3.1 Jejunal development *in vivo* and *ex vivo*

**Figure 1:**
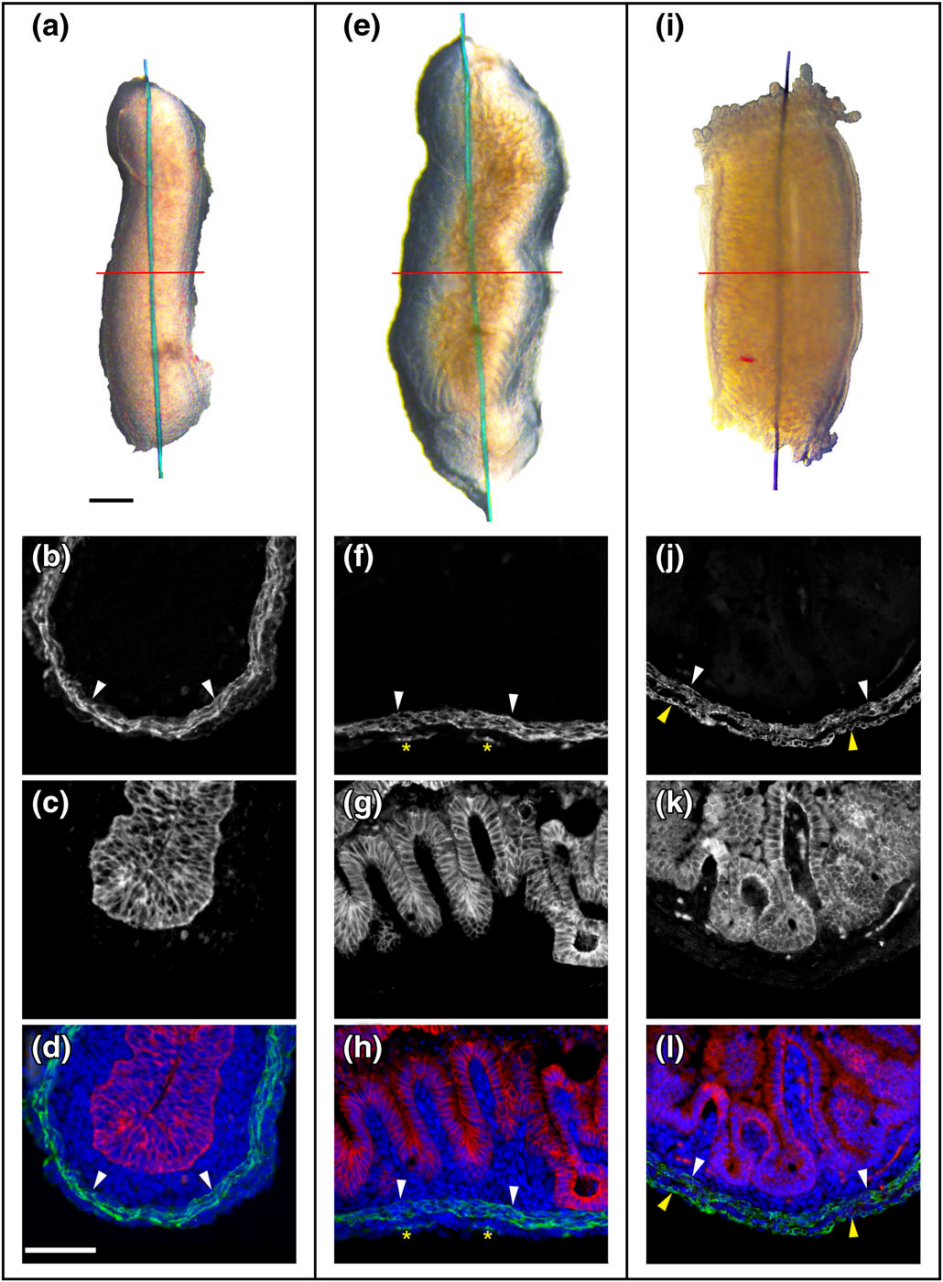
**Tissue layers in the embryonic jejunum.** A, E and I are gut segments, each kept straight by a suture threaded through the lumen. In each, the red line represents the plane of sectioning used to generate histology shown in B–D, F–H and J–L. (A) E14 jejunum on the day it was explanted. (B–E) Transverse section of this rudiment showing immunostaining for αSMA (white in B and green in the merged image in D) and E‐cadherin (white in C and red in the merged image in D). White arrowheads depict the circular SM layer. Note that there is no longitudinal SM layer. (E–H) Similar sequence for the floor of an E14 rudiment cultured for 3 days. Note the formation of villi and the presence of a circular SM layer; outside the latter, only a few scattered cells (yellow asterisks) express αSMA. (I–L) Similar sequence for a quadrant of a freshly dissected E17 jejunal segment. Note the presence of villi and both a circular and longitudinal (yellow arrowheads) layer. Scale bars are 250 μm. [Colour figure can be viewed at http://wileyonlinelibrary.com]

The tissue layers in the experimental model were first clarified. Freshly dissected E14 jejunum appeared as a semitranslucent cylinder (Figure 1A). On histology (Figure 1B–D), it contained an epithelial core immunostaining for E‐cadherin. This multilayered epithelium had indentations on its apical surface but lacked villi. The epithelial tube was surrounded by three sequential layers: mesenchymal‐like cells, a circular SM layer that immunostained for αSMA, and finally another mesenchymal‐like layer. Over 3 days in culture, and fed with basal media alone, the E14 explant increased in length and width (Figure 1E). A transverse section revealed a prominent ‘floor zone’ (Figure 1E, F) adjacent to the platform. This zone contained villi accounting for the ridge‐like pattern evident in whole mounts. Inner mesenchyme, circular SM, and outer mesenchyme were present in the floor. Although scattered cells in the outer mesenchyme expressed αSMA, typically a robust longitudinal SM layer was absent. By contrast, the explant *roof*, abutting air, was a simple structure lacking villi. Floor and roof zones were continuous and enclosed a lumen. A freshly dissected E17 jejunum is shown in Figure 1I. A transverse section (Figure 1J–L) revealed villi, an inner (circular) SM layer and an outer (longitudinal) SM layer. These observations show that, in organ culture, rudiments undergo a degree of differentiation and contain epithelial, mesenchymal and muscle cells.

### 3.2 Explant growth

**Figure 2:**
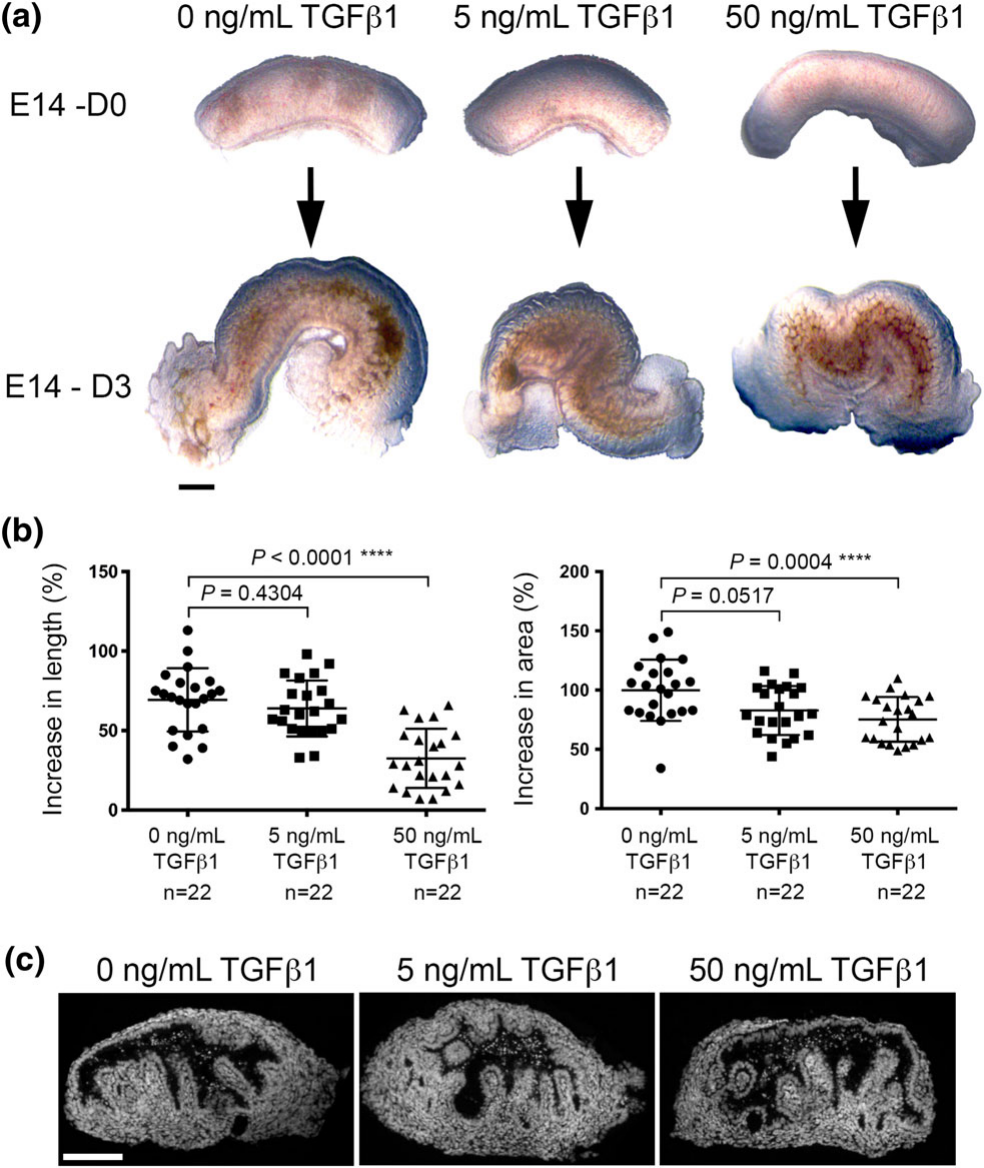
Effects of TGFβ1 on explant growth. (A) Upper images are three rudiments when they were explanted (E14–D0). They were respectively fed basal media alone, or media supplemented with 5 or 50 ng/mL TGFβ1. As depicted in the lower images, all three organs grew over 3 days. (B) Increases in length (left part of the frame) and area (right part of frame). The higher concentration of TGFβ1 retarded growth. Bars are mean ± SD. (C) Transverse sections of rudiments maintained for 3 days in organ culture, with nuclei stained (white) with DAPI. Scale bar is 250 μm. [Colour figure can be viewed at http://wileyonlinelibrary.com]

It was determined whether supplementing media with TGFβ1 altered growth by measuring explant length and area over 3 days in culture (Figure 2A). To account for small variations in starting sizes, percentage increases were calculated for each rudiment (Figure 2B). In basal media alone there was an average 69.3% increase in length and a 99.9% increase in area. Organs exposed to 5 or 50 ng/mL TGFβ1 also increased in size. With 5 ng/mL TGFβ1, there was no significant difference in length vs. rudiments fed basal media alone, although their areas were modestly decreased (*p =* 0.0517). By contrast, rudiments exposed to 50 ng/mL TGFβ1 had marked and significant decreases in growth vs. controls in both lengths and areas. Despite these concentration‐dependent effects of TGFβ1 on overall growth, in all three experimental conditions organs contained a floor and a roof, as assessed by histology (Figure 2C).

### 3.3 Effects of TGF**β** on embryonic muscle layers

**Figure 3:**
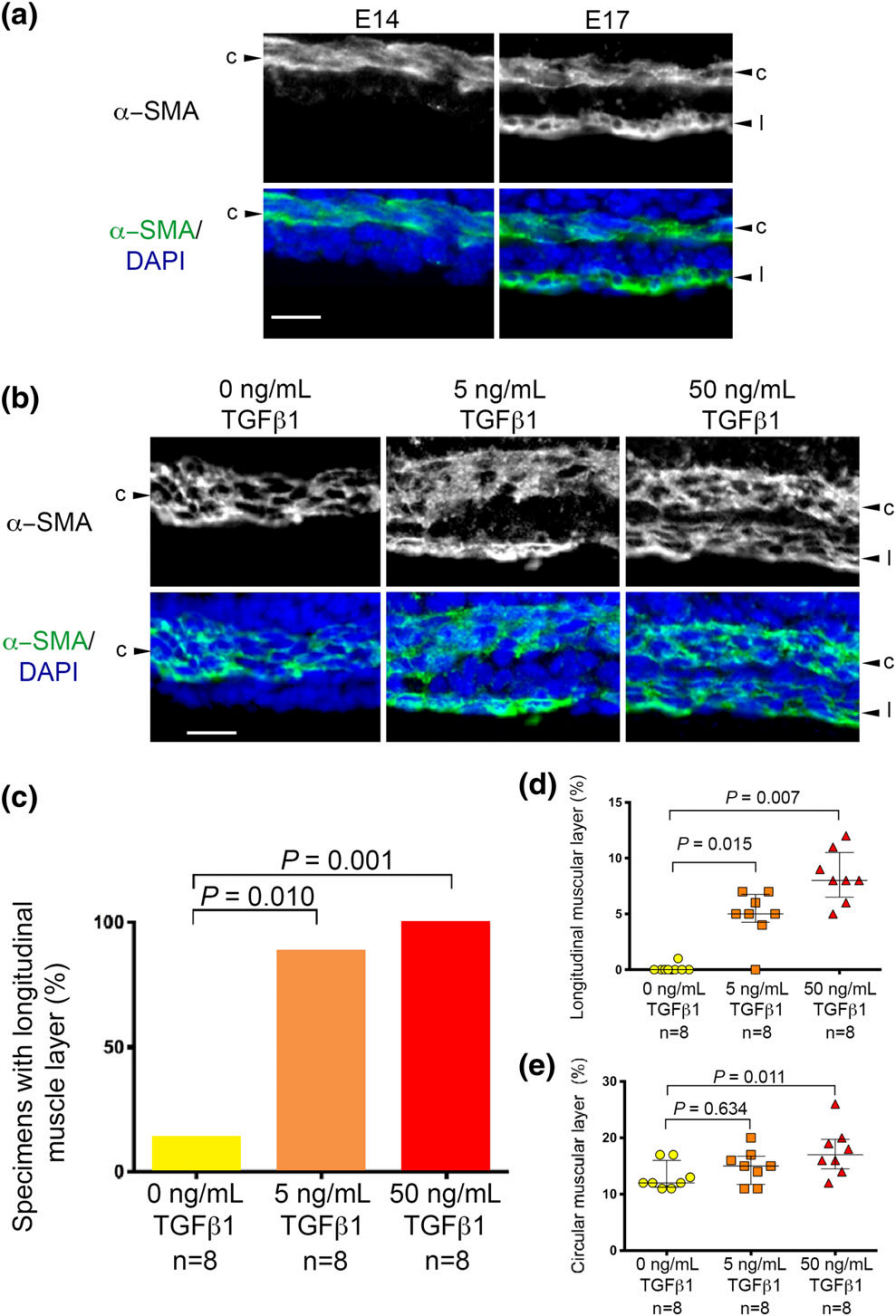
**Muscle in embryonic jejunum.** (A) High power images of transverse sections of freshly isolated intestines (E14 and E17 *in vivo*). Upper frames show αSMA immunostaining (white); lower fames are the same sections with DAPI stained nuclei (blue) and αSMA in green and nuclei in blue. Note that a circular SM layer (*c*) is present at both times but only the E17 organ contains a longitudinal (*l*) layer. (B) Similar sequences for explants fed basal media alone (left panels), or media supplemented with 5 (middle panels) or 50 (right panels) ng/mL TGFβ1. Note that longitudinal muscle is only present in the latter two conditions. Scale bar is 10 μm. (C–E) Data from eight sets of organ cultures. Each comprised rudiments from three embryos in the same litter, one explant fed basal media alone, and littermate rudiments fed 5 or 50 ng/mL TGFβ1. (C) Proportions of explants containing a longitudinal SM layer. (D) Percentage areas of explant floors (bars indicate medians and interquartile ranges) occupied by longitudinal SM. (E) Percentage areas (bars indicate medians and interquartile ranges) occupied by circular SM. [Colour figure can be viewed at http://wileyonlinelibrary.com]

Next, the detailed patterns of αSMA immunostaining within rudiments were considered, comparing organ cultures with freshly isolated E14 and E17 jejuna. Transverse sections of E14 organs (Figure 3A, left panel) showed a band of circular SM two or three cells thick but no longitudinal muscle layer. By contrast, E17 organs *in vivo* contained both a circular and a longitudinal SM layer (Figure 3A, right panel). Figure 3B shows representative histology images of organs cultures grown for 3 days in basal media alone (left panel), 5 ng/mL TGFβ1 (middle panel) and 50 ng/mL TGFβ1 (right panel). Note that only the latter two have a longitudinal muscle layer. Only one of eight explants in basal media alone had a discrete longitudinal muscle layer, whereas seven of eight explants in 5 ng/mL TGFβ1, and all eight explants in 50 ng/mL TGFβ1, contained longitudinal SM (respectively, *p =* 0.01 and *p =* 0.001 vs. explants in basal media; Figure 3C). The two TGFβ1 concentrations caused step‐wise increases in percentages of the total floor area occupied by longitudinal SM (Figure 3D). All rudiments contained circular SM, and 50 ng/mL TGFβ1 significantly increased the proportion of the floor occupied by this layer (Figure 3E). The percentage area of transverse sections occupied by muscle layers in freshly dissected E17 organs (*n =* 8) were: longitudinal SM (6%, 5–7%, median and interquartile range) and circular SM (13%, 12–15%). The value for longitudinal SM was similar (*p =* 0.293) in E17 organs and explants exposed to 5 ng/ml TGFβ1, while it was increased (*p =* 0.032) in explants exposed to 50 ng/ml TGFβ1 vs. E17 organs. Moreover, the value for circular SM was similar (*p =* 0.331) in E17 organs and explants exposed to 5 ng/ml TGFβ1, while it was increased (*p =* 0.028) in explants exposed to 50 ng/ml TGFβ1 vs. E17 organs.

### 3.4 Detection of TGF**β** receptors

**Figure 4:**
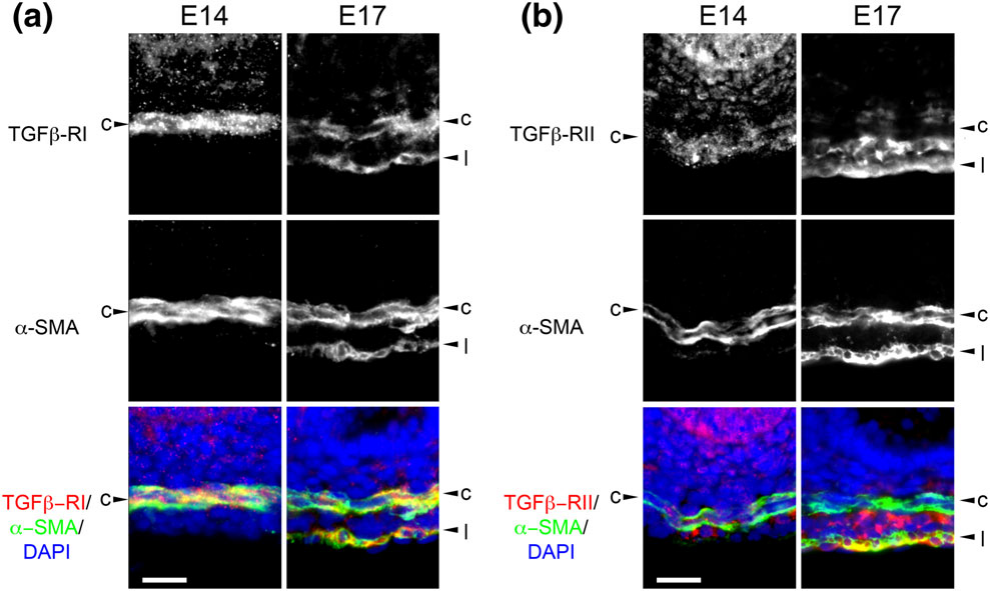
**Immunohistochemistry for TGFβ receptors.** Transverse histology sections of freshly isolated jejuna (E14 and E17 *in vivo*). (A) Frames in the top row show TGFβ‐R1 immunostaining (white); middle row shows the same sections immunostained for αSMA (white); the bottom row shows merged images with TGFβ‐RI in red, αSMA in green, and nuclei in blue. This receptor was detected in circular muscle (c) at E14 and in this and also the longitudinal (l) layer at E17. Note that longitudinal muscle is absent at E14 (B) Similar sequences immunostained for TGFβ‐RII. TGFβ‐RII was detected in circular muscle at E14, and in both longitudinal muscle and the mesenchyme between the two muscle layers at E17. Scale bars are 20 μm. [Colour figure can be viewed at http://wileyonlinelibrary.com]

*In vivo*, at E14, TGFβ‐RI was detected in the circular SM muscle, and, at E17, it was detected in circular and longitudinal SM layers (Figure 4A). With regard to TGFβ‐RII, at E14 it was detected in the circular SM muscle (Figure 4B). However, at E17, it was most prominent in the longitudinal SM layer and in the mesenchyme between the two muscle layers (Figure 4B). TGFβ proteins initiate signalling after binding to TGFβ receptors I and II (TGFBRI and II) (Akhurst and Hata 2012). Therefore, longitudinal muscle has the appropriate receptors to respond to TGFβ1.

### 3.5 The jejunal transcriptome

**Figure 5:**
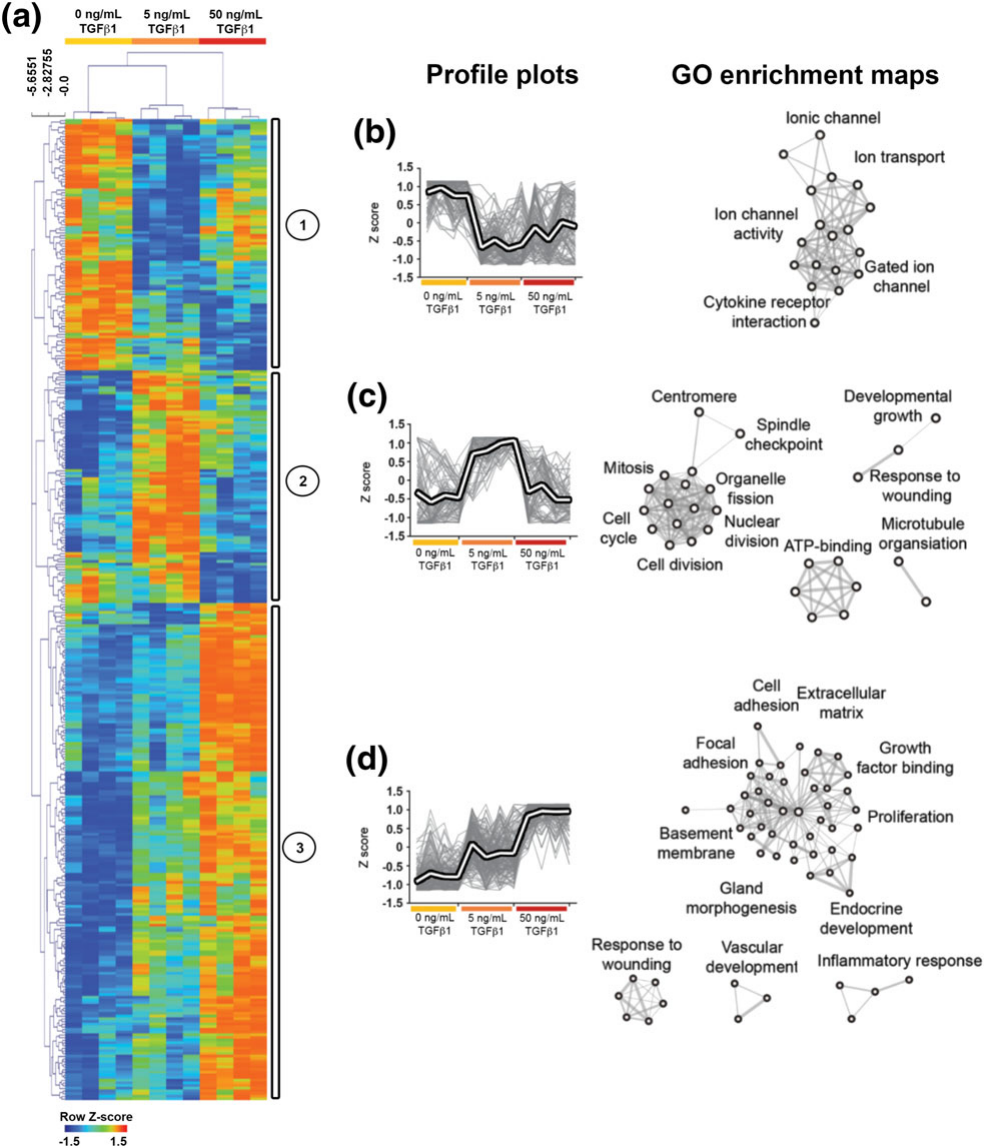
**Microarray analysis reveals TGFβ1 dose dependent effects.** A. Unsupervised hierarchical clustering by transcript expression. Rows are expression levels denoted as the z‐score, displayed in a high‐low (red‐blue) colour scale. Transcripts grouped into three Clusters: (1) reduced in response to TGFβ1, (2) increased in response to 5 ng/mL TGFβ1 and (3) increased in response to 50 ng/mL TGFβ1. Profile plots of these respective clusters are shown in B–D, with the mean profile for each cluster indicated by a white line. Over‐represented biological terms from each cluster are displayed as GO enrichment maps. Nodes represent GO terms and lines represent the degree of overlap. Cluster 1 revealed ion channels and membrane transcripts that were underrepresented following treatment with TGFβ1. Cluster 2 revealed cell‐cycle and microtubule process transcripts that were overrepresented following 5 ng/mL TGFβ1. Cluster 3 revealed transcripts involved in ECM and focal adhesion type processes that were overrepresented after exposure to TGFβ1. [Colour figure can be viewed at http://wileyonlinelibrary.com]

**Figure 6:**
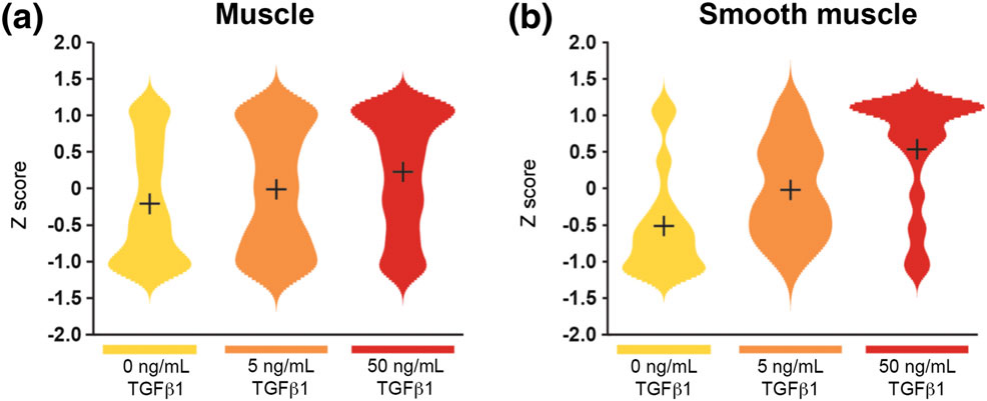
**Muscle transcripts.** Violin plots of z‐scores show levels of transcripts along vertical axes. (A) General muscle transcripts showed little change with TGFβ1. (B) SM specific transcripts increased in a stepwise manner with increasing TGFβ1 concentrations. [Colour figure can be viewed at http://wileyonlinelibrary.com]

To define molecular changes elicited by TGFβ1 in an unbiased manner, microarray analyses on organ culture samples were undertaken (Figure 5). In those exposed to 5 ng/mL TGFβ1, 175 transcripts were increased and 130 were decreased >1.4 fold. In 50 ng/mL TGFβ1, 246 transcripts were increased and 75 were decreased >1.4 fold. The most deregulated transcripts are shown in Tables 1 and 2, with the full list available at ArrayExpress database (http://www.ebi.ac.uk/arrayexpress accession number E‐MTAB‐450) and are shown in the Venn diagrams in Figure S1. We validated the microarray data with QPCR for 13 of the transcripts in the 5 ng/ml TGFβ1 cultures (Figure S2). Ingenuity pathway analysis confirmed the observed changes were consistent with activated TGFβ1 pathways. In rudiments exposed to 5 ng/mL TGFβ1, there were *TGFβ1 growth factor‐related changes* (*p‐*value of overlap, 2.81 × 10^–6^; activation z‐score, 2.50), and *SMAD3‐related changes* (*p‐*value of overlap, 6.75 × 10^–4^; activation z‐score, 2.38). The latter indicates activation of the canonical TGFβ signalling pathway (Akhurst and Hata 2012). In rudiments exposed to 50 ng/mL TGFβ1, there were *TGFβ1 growth factor‐related changes* (*p‐*value of overlap, 2.07 × 10^–22^; activation z‐score, 3.91) and *P38 mitogen activated protein kinase (MAPK)‐related changes* (*p‐*value of overlap, 1.02 × 10^–10^; activation z‐score, 3.67). The latter indicates activation of a SMAD independent/noncanonical signalling TGFβ pathway (Akhurst and Hata 2012). Unsupervised hierarchical clustering revealed three major gene expression patterns, Clusters 1–3. Cluster 1 comprised 90 unique transcripts with reduced expression in explants exposed to exogenous TGFβ1. Gene ontology enrichment analysis revealed that most encoded transmembrane proteins, including ion channels. Cluster 2 contained 66 transcripts increased in explants exposed to 5 ng/mL TGFβ1, but not in those exposed to 50 ng/mL TGFβ1 (Figure 5C). These transcripts prominently encoded proteins involved in the cell cycle. Cluster 3 contained 170 increased transcripts with the highest expression in explants exposed to 50 ng/mL TGFβ1. They prominently encoded extracellular matrix (ECM), cellular adhesion and focal adhesion proteins. Using the Jackson Laboratory MGI‐Mouse gene expression database, we analysed tissue specific transcript sets (Figure 6). SM markers displayed a TGFβ1 concentration‐dependent increase, whereas less specific muscle markers were only marginally increased. Collectively, these results demonstrate complex sets of changes in gene expression in response to exogenous TGFβ1, with both concentration‐dependent and concentration‐specific effects.

**Table 1:** The most deregulated transcripts in organs exposed to 5 ng/ml TGFβ1

**UniGene Symbol**	**UniGene Name**	**Fold change**	***p***
Upregulated in 5 ng/ml TGFβ1 vs. basal media			
*Car6*	Carbonic anhydrase 6	3.02	0.02
*Pigr*	Polymeric immunoglobulin receptor	3.01	0.01
*Anxa10*	Annexin A10	2.89	0.01
*Ifit1*	Interferon‐induced protein with tetratricopeptide repeats1	2.64	0.04
*Ctsw*	Cathepsin W	2.27	0.0001
*Mfap5*	Microfibrillar associated protein 5	2.11	0.002
*Col8a1*	Collagen, type VIII, α1	2.10	0.0001
*Krt17*	Keratin 17	2.07	0.0006
*Lgr5*	Leucine‐rich repeat containing G protein‐coupled receptor 5	1.90	0.004
*Eln*	Elastin	1.82	0.005
Downregulated in 5 ng/ml TGFβ1 vs. basal media			
*Cdh16*	Cadherin 16	–3.80	0.03
*Dppa5a*	Developmental pluripotency associated 5a	–2.89	0.02
*Akp3*	Alkaline phosphatase 3, intestine, not Mn requiring	–2.63	0.04
*Fxyd2*	FXYD domain containing ion transport regulator 2	–2.53	0.04
*Paqr5*	Progestin and adipoQ receptor family member V	–2.41	0.04
*Tufm*	Tu translation elongation factor, mitochondrial	–2.33	0.01
*Hsd3b2*	Hydroxyl‐δ‐5‐steroid dehydrogenase, 3 β‐ and steroid δ‐isomerase 2	–2.31	0.02
*Agmo*	Alkylglycerosl monooxygenase	–2.25	0.03
*Adam4*	A disintegrin and metallopeptidase domain 4	–1.78	0.001

**Table 2:** The most deregulated transcripts in organs exposed to 50 ng/ml TGFβ1

**UniGene Symbol**	**UniGene Name**	**Fold change**	***p***
Upregulated in 50 ng/ml TGFβ1 vs. basal media			
*C1qtnf3*	C1q and tumour necrosis factor‐related protein 3	5.68	1.69 × 10^–5^
*Adamts16*	A disintegrin‐like and metallopeptidase with thrombospondin type 1, motif 16	3.93	5.67 × 10^–6^
*Akr1c18*	Aldo‐keto reductase family 1, member C18	3.73	0.0006
*Adamtsl2*	ADAMTS‐like2	3.10	1.53 × 10^–5^
*Ibsp*	Integrin binding sialoprotein	3.10	0.003
*Mfap5*	Microfibrillar associated protein 5	3.08	0.0001
*Cilp*	Cartilage intermediate layer protein, nucleotide pyrophosphohydrolase	3.02	0.007
*Ctsw*	Cathepsin W	2.96	4.97 × 10^–5^
*Col8a1*	Collagen, type VIII, α1	2.88	0.0004
*Eln*	Elastin	2.73	2.18 × 10^–5^
***Downregulated in 50 ng/ml TGFβ1* vs. *basal media***			
*Gpm6a*	Glycoprotein m6a	–2.87	3.95 × 10^–5^
*Dppa5a*	Developmental pluripotency associated 5a	–2.50	0.03
*Tufm*	Tu translation elongation factor, mitochondrial	–2.21	0.0008
*Gpx2*	Glutathione peroxidase 2	–2.13	0.01
*Kcne3*	Potassium voltage‐gated channel. Isk‐related family, member 3	–1.80	0.002
*Kcnn3*	Potassium intermediate/small conductance calcium‐activated channel, subfamily N, member 3	–1.79	0.01
*Upk1b*	Uroplakin 1B	–1.77	0.0004
*Pten*	Phosphatase and tensine homolog	–1.76	0.01
*Celf1*	CUG triplet repeat, Elav‐like family member 1	–1.69	0.005
*Sema3d*	Sema domain, immunoglobulin domain, short basic domain, secreted 3D	–1.62	0.02

### 3.6 Proliferation in explants

Given that the microarray data showed an increase in cell cycle transcripts following exposure to 5 ng/mL TGFβ1, cell number and proliferation were analysed in tissue sections. Floors of rudiments exposed to 5 ng/mL TGFβ1 tended to have more nuclei (*p =* 0.07) than controls. Proliferation was most prominent in mesenchymal and longitudinal muscle layers in the rudiments exposed to 5 ng/mL TGFβ1 (data not shown).

### 3.7 TGF**β** in jejunum and amniotic fluid

**Figure 7:**
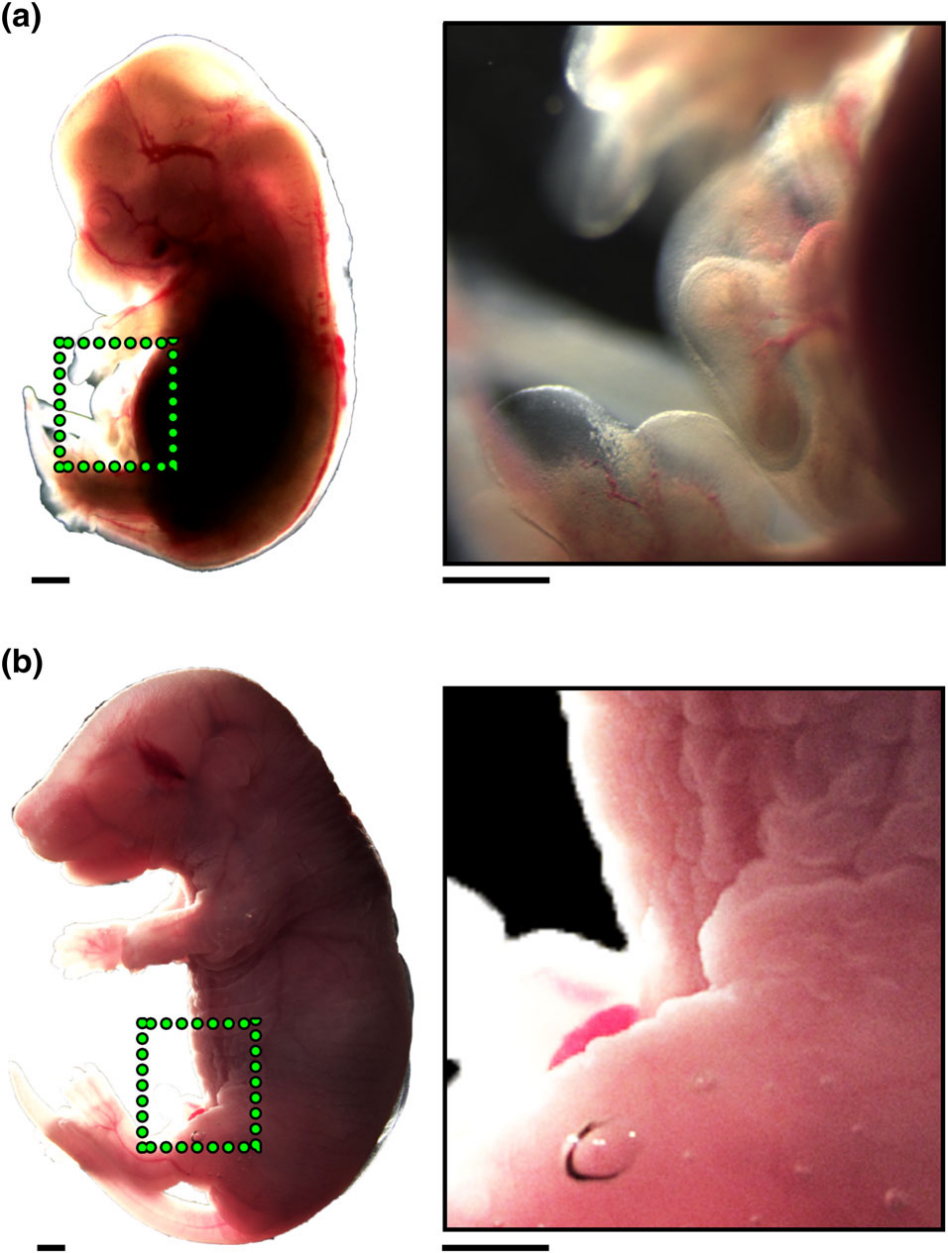
**Embryo whole mounts.** (A) Image on left is a whole E14 embryo and inset on right is a high‐power image of the boxed area showing intestine protruding into the extraembryonic coelom. (B) Image on left is a whole E17 embryo and inset on right is a high‐power image of the boxed area showing that the intestine has returned into the body cavity. [Colour figure can be viewed at http://wileyonlinelibrary.com]

In the E14 embryo, herniated intestine protruded into the extraembryonic coelom of the umbilicus (Figure 7A). At E17 *in vivo*, the intestine had returned into the abdominal cavity (Figure 7B). Note that the intestine had a close spatial relation with the amniotic fluid. Amniotic fluid contained TGFβ1 at E14 [1.39 ± 0.31 ng/mL; mean ± standard deviation (SD), *n =* 3] and E17 (2.73 ± 1.72 ng/mL, *n =* 3), with no significant difference (*p =* 0.114) in concentrations between these stages. Next, TGFβ1 was sought in the gut itself. As assessed by QPCR both the E14 and E17 jejunum expressed *Tgfβ1*, with levels falling between these embryonic stages (Figure S3). E14 explants, cultured for 3 days with basal media alone, contained levels of *Tgfβ1* transcripts similar to E17 organs *in vivo*. Despite the detection of *Tgfβ1* transcripts in explants, there was no detectable TGFβ1 protein in organ culture conditioned media (lower limit of detection being 0.03 ng/mL).

## 4 Discussion

### 4.1 TGF**β**1 and jejunal muscle differentiation

The current study reveals marked effects of exogenous TGFβ1 on the muscularization of embryonic small intestine. RNA microarrays of cultured organs showed that TGFβ1 increased SM specific transcripts in a concentration‐dependent manner. Moreover, detailed examination of histology sections within explants revealed that exogenous TGFβ1 increased the proportions of the tissue occupied by SM. We found that in the embryonic mouse small intestine, as reported in rats (Kedinger et al., 1990), circular SM is present before longitudinal SM has formed. Strikingly, in the current *ex vivo* model, exogenous TGFβ1 was essential for differentiation of longitudinal SM. Moreover, the detection of TGFβ receptors I and II in differentiating muscle cells is consistent with a direct effect of TGFβ1 in this process.

Diverse growth factors have been implicated in controlling gut differentiation, as assessed by experiments in diverse vertebrate species (Rubin 2007), and our results should be considered in relation to this body of knowledge. Embryonic gut endodermal cells secrete sonic hedgehog (SHH) that stimulates visceral mesoderm to form SM (Apelqvist et al., 1997). Bone morphogenetic protein, an SHH target (Roberts et al., 1995), and platelet derived growth factor (PDGF) are expressed in the gut wall where SM is forming and have themselves been functionally implicated in SM differentiation (Kurahashi et al., 2008; Torihashi et al., 2009). As well as being a signaling centre for SHH, embryonic intestinal epithelia secrete FGF9 that drives gut growth, enhancing mesenchymal proliferation (Geske et al., 2008). In this context, genetic deletion of *Fgf9* led to premature gut muscularization in association with upregulated TGFβ signaling (Geske et al., 2008).

In most of the above studies, gut SM was considered as a single entity. An exception is the report by Kurahashi et al. (2008) that concluded that PDGF‐A is expressed by circular SM, and that PDGF‐B is expressed by interstitial cells of Cajal, and that these factors acted on longitudinal SM precursor cells expressing PDGFRα and β. In our RNA array, we found a significant upregulation of *Pdgfa* (1.33‐fold change in 5 ng/mL and 1.22 in 50 ng/mL TGFβ1) and *Pdgfc* (1.38 fold change in 5 ng/mL and 1.66 in 50 ng/mL TGFβ1). There was no significant change in *Fgf9* transcripts after exposure to 5 ng/mL TGFβ1 but in rudiments exposed to 50 ng/mL TGFβ1 *Fgf9* levels were significantly lower (0.82) than controls. Thus, as well as having a direct effect of longitudinal SM differentiation, TGFβ1 may indirectly enhance embryonic intestinal muscle differentiation *via* alterations of these growth factors. No significant changes were noted in *Pdgfb*, *Pdgfd* or the receptors *Pdgfra* and *Pdgfrb*, nor in the HH pathway transcripts *Shh*, *Ptch1*, *Ptch2*, *Bmp2* and *Bmp4*.

### 4.2 Sources of TGF**β** *in vivo*

Whereas longitudinal muscle differentiated in the small intestine *in vivo* between E14 and E17, it failed to do so in explants fed basal media alone. Strikingly, however, longitudinal SM differentiation was rescued by the addition of exogenous TGFβ1. As assessed by QPCR, *Tgfb1* transcripts were detected in the embryonic jejunum, with levels tending to fall between E14 and E17. Moreover, these transcripts, at levels similar to those found at E17 *in vivo*, were detected in E14 jejunal rudiments cultured for 3 days. By contrast, conditioned media in of explants fed basal media alone did not contain detectable levels of TGFβ1. We found at mouse E14, anatomically equivalent to week 7 of human gestation (Sadler, 1990), the elongating intestine protrudes into the extraembryonic celom of the umbilicus where it is in close proximity to the amniotic cavity. By mouse E17, anatomically equivalent to week 9 of human gestation (Sadler, 1990), the small intestine has become tightly coiled and has returned to the body cavity. Amniotic fluid has previously been shown to contain TGFβ, at least some of which is derived from the mother (Letterio et al., 1994; McLennan and Koishi, 2004). In the current study, we showed that amniotic fluid contained TGFβ1 in concentrations of about 1–2 ng/mL, at a time when the intestine is physiologically herniated. Thus, by analogy with the organ culture results, we suggest that fluid bathing the intestine may enhance its differentiation *in vivo*. This concept, that exogenous TGFβ supplements endogenous gut TGFβ, is similar to one proposed by Penttila et al. (1998) who found that maternal milk was rich in this growth factor. A recent mouse study by Zhang et al. (2016) used QPCR to measure levels of a variety of growth factor receptors in small intestine between E13 and postnatal day 60. They found that transcripts encoding TGFβ‐RI were highest at E13, and fell later in gestation, then rose to a smaller peak at 2 weeks after birth. Transcripts encoding TGFβ‐RII rose between E13 and 2 weeks after birth, and then decreased. The study reported that milk contained an average of 900 ng/ml of TGFβ1 at birth, with levels of TGFβ2 approximately an order of magnitude less (Zhang et al., 2016). In the first few postnatal weeks, the authors contended that TGFβ proteins in milk may affect epithelial terminal differentiation, and play an anti‐inflammatory effect, in the small intestine.

### 4.3 Optimal levels of TGF**β**1 *in vivo*

While TGFβ1 appeared to facilitate jejunal development, especially in relation to muscularization, our results point to the additional conclusion that the higher TGFβ1 concentration had additional, deleterious effects. While exposure of E17 explants to 5 ng/ml TGFβ1 for 3 days produced a pattern of SM layering similar to freshly dissected E17 organs, as assessed by percentage of explant areas occupied by αSMA immunostaining, the addition of 50 ng/ml TGFβ1 appeared to generate an overabundance of SM vs. E17 organs. Scrutiny of the array data revealed that some transcripts were upregulated by between 50 and 5 ng/mL TGFβ1 that were unchanged between 5 and 0 ng/mL TGFβ1. They included metalloproteases *Adamts16*, *Adamts20*, a skeletal collagen *Col24a1*, integrin *Itga9* and laminin *Lama1*. Furthermore, the array indicated that SMAD independent/noncanonical TGFβ signalling became activated at the higher concentration of TGFβ1.

In terms of increases in explant areas and lengths over 3 days in culture, the higher concentration (50 ng/ml) of TGFβ caused an overall slowing of growth. Yamada et al. (2013) noted that exogenous TGFβ1 inhibited the proliferation of the rat IEC‐6 intestinal epithelial cell line in a dose‐dependent manner. In the current study, we found a borderline significant (*p =* 0.062) decrease in proliferation of epithelia in embryonic jejunal explants exposed to the higher concentration (50 ng/ml) of TGFβ1. These retardation effects recall those reported in embryonic organ cultures of mouse kidneys and salivary glands exposed to exogenous TGFβ (Clark et al., 2001; Hardman et al., 1994; Rogers et al., 1993). Here, there may be a parallel with a variety of human diseases in which excessive TGFβ signalling has been associated with aberrant development and differentiation. As examples, TGFβ deregulation has been implicated in metaplastic SM formation within malformed kidneys (Yang et al., 2000), aberrant ECM modelling within aortic aneurysms in Marfan syndrome (Nataatmadja et al., 2006) and the formation of fibrotic strictures in Crohn's disease (Li et al., 2015). One could also speculate that in the malformation called gastroschisis, where herniated gut fails to return to the abdominal cavity, the intestine would suffer overexposure to growth factors in the amniotic including TGFβ1.

Our current study cannot answer the question of whether *Tgfb1* transcripts expressed in the embryonic intestine play a biological role in gut development. TGFβ1 null mutant die either at E10–11 with defects in yolk sac vasculature (Dickson et al., 1995), or in the first few postnatal weeks where they suffer a wasting disorder accompanied by multiorgan inflammation and *mild colonic and gastric necrosis* (Kulkarni et al., 1993). A similar embryonic phenotype was observed in homozygous *Tgfbr2* mutant mice (Oshima et al., 1996) and *Tgfbr2/wild type* chimeric mice progressed through the fetal period but suffered postnatal wasting accompanied and their *organs were smaller* than normal. Further studies may be more informative if TGFβ1 or its receptors could be experimentally downregulated in specific tissue layers and sections of the developing gut.

## 5 Conclusions

This *ex vivo* study reveals striking effects of TGFβ1 on small intestinal muscularization. By analogy with the requirement for exogenous TGFβ1 in organ culture experiments, TGFβ1 protein normally present in the amniotic fluid may enhance development of the gut when it is physiologically herniated in early gestation. Future studies of embryonic intestinal cultures should include TGFβ1 in the defined media to produce a more faithful model of *in vivo* muscle differentiation. Finally, it is notable that human pluripotent stem cells can be induced to form intestinal organoids in culture (Wells and Spence, 2014). While most attention has been given to epithelial morphogenesis in this model, the organoids can also contain mesenchyme that forms SM and fibroblasts. In future, it will be informative to determine whether TGFβ affects the differentiation of these mesenchyme‐derived cells.

### Project sponsors

These studies were supported by the Short Bowel Survivor and Friends Charity, the Medical Research Council (MR/L002744/1 and MR/K026739/1) and Kidney Research UK. The work was facilitated by the Manchester Biomedical Research Centre and the Greater Manchester Comprehensive Local Research Network.

## Author contributions

R.C., N.A.R. and A.S.W. designed the study, interpreted organ culture results and wrote the body of the paper. R.C. undertook the organ culture experiments. M.J.R. undertook bioinformatic analyses of microarrays. A.M. helped to supervise the study and obtained funding. All authors approved the final manuscript.

## Conflict of interest

The authors have no conflict of interest to disclose.

## Abbreviations

E, embryonic day; QPCR, quantitative polymerase chain reaction; TGFβ, transforming growth factor β; TGFβR, TGFβ receptor; αSMA, α‐smooth muscle actin.

## Supporting information


**Data S1. Supporting info item**
**Supplemental Figure S1. Venn diagrams showing the significantly up and down regulated transcripts in organ cultures administered TGFβ1 vs. explants exposed to basal media alone.** The number in parentheses next to each concentration of TGFβ1 is the total up‐ or down‐regulated transcripts for that concentration. The numbers of transcripts in the Overlaps (i.e. changed in both concentration of TGFβ1 vs. basal media) are also indicated.
**Supplemental Figure S2. Comparisons of changes in levels of selected transcripts measured by microarray and QPCR**. Changes in levels of transcripts as assessed by microarray (blue) and QPCR (red) analyses. Each column is the mean ( ± SD) of four experimental replicates of organs exposed to 5 ng/mL TGFβ1 vs. organs exposed to basal media alone. Note that the patterns generated by the two analytical methods are similar.
**Supplemental Figure S3. Expression of *Tgfb1* in embryonic jejunum *in vivo* and in organ culture.** QPCR measurements for *Tgfb1* factored for *Gapdh*, expressed as the mean ± SD of three samples of freshly dissected E14 jejunum (yellow, E14–D0), freshly dissected E17 jejunum (orange, E17), and E14 rudiments cultured for 3 days in basal media alone (red, E14‐D3). Levels of *Tgfβ1* transcripts fell (*p =* 0.01) when comparing E14 and E17 jejuna and also when comparing freshly dissected E14 organs with those cultured for 3 days

## References

[term2409-bib-0001] Akhurst RJ , Hata A . 2012; Targeting the TGFβ signalling pathway in disease. Nat Rev Drug Discov 11: 790–811. 23000686 10.1038/nrd3810 PMC3520610

[term2409-bib-0002] Apelqvist A , Ahlgren U , Edlund H . 1997; Sonic hedgehog directs specialised mesoderm differentiation in the intestine and pancreas. Curr Biol 7: 801–804. 9368764 10.1016/s0960-9822(06)00340-x

[term2409-bib-0003] Baldi P , Long AD . 2011; A Bayesian framework for the analysis of microarray expression data: regularized *t*‐test and statistical inferences of gene changes. Bioinformatics 17: 509–519. 10.1093/bioinformatics/17.6.509 11395427

[term2409-bib-0004] Barnard JA , Warwick GJ , Gold LI . 1993; Localization of transforming growth factor β isoforms in the normal murine small intestine and colon. Gastroenterology 105: 67–73. 8514063 10.1016/0016-5085(93)90011-z

[term2409-bib-0005] Cervantes S , Yamaguchi TP , Hebrok M . 2009; Wnt5a is essential for intestinal elongation in mice. Dev Biol 326: 285–294. 19100728 10.1016/j.ydbio.2008.11.020 PMC2654720

[term2409-bib-0006] Clark AT , Young RJ , Bertram JF . 2001; *In vitro* studies on the roles of transforming growth factor‐β1 in rat metanephric development. Kidney Int 59: 1641–1653. 11318934 10.1046/j.1523-1755.2001.0590051641.x

[term2409-bib-0007] Coletta R , Khalil BA , Morabito A . 2014; Short bowel syndrome in children: surgical and medical perspectives. Semin Pediatr Surg 23: 291–297. 25459014 10.1053/j.sempedsurg.2014.09.010

[term2409-bib-0008] Coletta R , Roberts NA , Oltrabella F et al. 2016; Bridging the gap: functional healing of embryonic small intestine *ex vivo* *.* J Tissue Eng Regen Med 10;178–182. 26234729 10.1002/term.2073 PMC4950007

[term2409-bib-0009] Dickson MC , Martin JS , Cousins FM et al*.* 1995 Defective haematopoiesis and vasculogenesis in transforming growth factor‐beta 1 knock out mice. Development 121: 1845–1854. 7600998 10.1242/dev.121.6.1845

[term2409-bib-0010] Geske MJ , Zhang X , Patel KK et al. 2008; Fgf9 signaling regulates small intestinal elongation and mesenchymal development. Development 135: 2959–2968. 18653563 10.1242/dev.020453 PMC2678066

[term2409-bib-0011] Ghionzoli M , Repele A , Sartiani L et al. 2013; Human amniotic fluid stem cell differentiation along smooth muscle lineage. FASEB J 27: 4853–4865. 23995291 10.1096/fj.12-218578 PMC6188351

[term2409-bib-0012] Hall NJ , Eaton S , Pierro A . 2013; Royal Australasia of Surgeons Guest Lecture. Necrotizing enterocolitis: prevention, treatment, and outcome. J Pediatr Surg 48: 2359–2367. 24314171 10.1016/j.jpedsurg.2013.08.006

[term2409-bib-0013] Hardman P , Landels E , Woolf AS et al. 1994; Transforming growth factor‐β1 inhibits growth and branching morphogenesis in embryonic mouse submandibular and sublingual glands *in vitro* . Develop Growth Differ 36: 567–577. 10.1111/j.1440-169X.1994.00567.x 37281074

[term2409-bib-0014] Huang da W , Sherman BT , Lempicki RA . 2009a; Systematic and integrative analysis of large gene lists using DAVID bioinformatics resources. Nat Protoc 4: 44–57. 19131956 10.1038/nprot.2008.211

[term2409-bib-0015] Huang da W , Sherman BT , Lempicki RA . 2009b; Bioinformatics enrichment tools: paths toward the comprehensive functional analysis of large gene lists. Nucleic Acids Res 37: 1–13. 19033363 10.1093/nar/gkn923 PMC2615629

[term2409-bib-0016] Huang WY , Xie W , Guo X et al. 2011; Smad2 and PEA3 cooperatively regulate transcription of response gene to complement 32 in TGF‐β‐induced smooth muscle cell differentiation of neural crest cells. Am J Physiol Cell Physiol 301: C499–C506. 21613609 10.1152/ajpcell.00480.2010 PMC3154553

[term2409-bib-0017] Kedinger M , Simon‐Assmann P , Bouziges F et al. 1990; Smooth muscle actin expression during rat gut development and induction in fetal skin fibroblastic cells associated with intestinal embryonic epithelium. Differentiation 43: 87–97. 2197142 10.1111/j.1432-0436.1990.tb00434.x

[term2409-bib-0018] Kulkarni AB , Huh CG , Becker D et al. 1993; Transforming growth factor beta 1 null mutation in mice causes excessive inflammatory response and early death. Proc Natl Acad Sci U S A 90: 770–774. 8421714 10.1073/pnas.90.2.770 PMC45747

[term2409-bib-0019] Kurahashi M , Niwa Y , Cheng J et al. 2008; Platelet‐derived growth factor signals play critical roles in differentiation of longitudinal smooth muscle cells in mouse embryonic gut. Neurogastroenterol Motil 20: 521–531. 18194151 10.1111/j.1365-2982.2007.01055.x

[term2409-bib-0020] Letterio JJ , Geiser AG , Kulkarni AB et al. 1994; Maternal rescue of transforming growth factor‐beta 1 null mice. Science 264: 1936–1938. 8009224 10.1126/science.8009224

[term2409-bib-0021] Li C , Iness A , Yoon J et al. 2015; Noncanonical STAT3 activation regulates excess TGF‐β1 and collagen I expression in muscle of stricturing Crohn's disease. J Immunol 194: 3422–3431. 25740948 10.4049/jimmunol.1401779 PMC4369432

[term2409-bib-0022] Liu B , Feng D , Lin G et al. 2010; Signalling molecules involved in mouse bladder smooth muscle cellular differentiation. Int J Dev Biol 54: 175–180. 20013655 10.1387/ijdb.082610bl PMC2855152

[term2409-bib-0023] Maghsoudlou P , Urbani L , De Coppi P . 2014; Organ bioengineering for the newborn. Semin Pediatr Surg 23: 314–323. 25459018 10.1053/j.sempedsurg.2014.09.014

[term2409-bib-0024] Masumoto K , Suita S , Nada O et al. 1999; Abnormalities of enteric neurons, intestinal pacemaker cells, and smooth muscle in human intestinal atresia *.* J Pediatr Surg 34: 1463–1468. 10549748 10.1016/s0022-3468(99)90104-5

[term2409-bib-0025] McLennan IS , Koishi K . 2004; Fetal and maternal transforming growth factor‐β1 may combine to maintain pregnancy in mice. Biol Reprod 70: 1614–1618. 14766723 10.1095/biolreprod.103.026179

[term2409-bib-0026] Merico D , Isserlin R , Stueker O et al. 2010; Enrichment map: a network‐based method for gene‐set enrichment visualization and interpretation. PLoS One 5: e13984. 21085593 10.1371/journal.pone.0013984 PMC2981572

[term2409-bib-0027] Miyoshi H , Ajima R , Luo CT et al. 2012; Wnt5a potentiates TGF‐β signaling to promote colonic crypt regeneration after tissue injury. Science 338: 108–113. 22956684 10.1126/science.1223821 PMC3706630

[term2409-bib-0028] Nataatmadja M , West J , West M . 2006; Overexpression of transforming growth factor‐β is associated with increased hyaluronan content and impairment of repair in Marfan syndrome aortic aneurysm. Circulation 114(Suppl I): I371–I377. 16820603 10.1161/CIRCULATIONAHA.105.000927

[term2409-bib-0029] Oshima M , Oshima H , Taketo MM . 1996; TGF‐beta receptor type II deficiency results in defects of yolk sac hematopoiesis and vasculogenesis. Dev Biol 179: 297–302. 8873772 10.1006/dbio.1996.0259

[term2409-bib-0030] Penttila IA , van Spriel AB , Zhang MF et al*.* 1998; Transforming growth factor‐β levels in maternal milk and expression in postnatal rat duodenum and ileum. Pediatr Res 44: 524–531. 9773841 10.1203/00006450-199810000-00010

[term2409-bib-0031] Quackenbush J. 2001; Computational analysis of microarray data. Nat Rev Genet 2: 418–427. 11389458 10.1038/35076576

[term2409-bib-0032] Roberts DJ , Johnson RL , Burke AC et al. 1995; Sonic hedgehog is an endodermal signal inducing Bmp‐4 and Hox genes during induction and regionalization of the chick hindgut. Development 121: 3163–3174. 7588051 10.1242/dev.121.10.3163

[term2409-bib-0033] Roche KC , Gracz AD , Liu XF et al. 2015; SOX9 maintains reserve stem cells and preserves radioresistance in mouse small intestine. Gastroenterology 149: 1553–1563. 26170137 10.1053/j.gastro.2015.07.004 PMC4709179

[term2409-bib-0034] Rogers SA , Ryan G , Purchio AF et al. 1993; Metanephric transforming growth factor‐β1 regulates nephrogenesis *in vitro* . Am J Phys 264: F996–F1002. 10.1152/ajprenal.1993.264.6.F996 8322902

[term2409-bib-0035] Rubin DC . 2007; Intestinal morphogenesis. Curr Opin Gastroenterol 23: 111–114. 17268237 10.1097/MOG.0b013e3280145082

[term2409-bib-0036] Sadler TW . 1990; Digestive system In Langman's Medical Embryology, 6th ed Williams and Wilkens: Baltimore, MD: 237–259.

[term2409-bib-0037] Saeed AI , Sharov V , White J et al. 2003; TM4: a free, open‐source system for microarray data management and analysis. Biotechniques 34: 374–378. 12613259 10.2144/03342mt01

[term2409-bib-0038] Shannon P , Markiel A , Ozier O et al. 2003; Cytoscape: a software environment for integrated models of biomolecular interaction networks. Genome Res 13: 2498–2504. 14597658 10.1101/gr.1239303 PMC403769

[term2409-bib-0039] Storey JD , Tibshirani R . 2003; Statistical significance for genomewide studies. Proc Natl Acad Sci U S A 100: 9440–9445. 12883005 10.1073/pnas.1530509100 PMC170937

[term2409-bib-0040] Torihashi S , Hattori T , Hasegawa H et al. 2009; The expression and crucial roles of BMP signaling in development of smooth muscle progenitor cells in the mouse embryonic gut. Differentiation 77: 277–289. 19272526 10.1016/j.diff.2008.10.003

[term2409-bib-0041] Wales PW , Christison‐Lagay ER . 2010; Short bowel syndrome: epidemiology and etiology. Semin Pediatr Surg 19: 3–9. 20123268 10.1053/j.sempedsurg.2009.11.001

[term2409-bib-0042] Wells JM , Spence JR . 2014; How to make an intestine. Development 141: 752–760. 24496613 10.1242/dev.097386 PMC3912826

[term2409-bib-0043] Wilm B , Ipenberg A , Hastie ND et al. 2005; The serosal mesothelium is a major source of smooth muscle cells of the gut vasculature. Development 132: 5317–5328. 16284122 10.1242/dev.02141

[term2409-bib-0044] Yamada Y , Mashima H , Sakai T et al. 2013; Functional roles of TGF‐β1 in intestinal epithelial cells through Smad‐dependent and non‐Smad pathways. Dig Dis Sci 58: 1207–1217. 23306843 10.1007/s10620-012-2515-7

[term2409-bib-0045] Yang SP , Woolf AS , Yuan HT et al. 2000; Potential biological role of transforming growth factor‐1 in human congenital kidney malformations. Am J Pathol 157: 1633–1647. 11073823 10.1016/s0002-9440(10)64801-8 PMC3277215

[term2409-bib-0046] Zhang M , Liao Y , Lönnerdal B . 2016; Milk growth factors and expression of small intestinal growth factor receptors during the perinatal period in mice. Pediatr Res 80: 759–765. 27603563 10.1038/pr.2016.150

