# Supplementary material for: Exogenous transforming growth factor‐β1 enhances smooth muscle differentiation in embryonic mouse jejunal explants
Source: J Tissue Eng Regen Med. 2017 Apr 27;12(1):252–64. doi: 10.1002/term.2409 (PMC6485323; doi:10.1002/term.2409)
Supplement: Supplementary file 1 — Data S1. Supporting info item Supplemental Figure S1. Venn diagrams showing the significantly up and down regulated transcripts in organ cultures administered TGFβ1 vs. explants exposed to basal media alone. The number in parentheses next to each concentration of TGFβ1 is the total up‐ or down‐regulated transcripts for that concentration. The numbers of transcripts in the Overlaps (i.e. changed in both concentration of TGFβ1 vs. basal media) are also indicated. Supplemental Figure S2. Comparisons of changes in levels of selected transcripts measured by microarray and QPCR. Changes in levels of transcripts as assessed by microarray (blue) and QPCR (red) analyses. Each column is the mean ( ± SD) of four experimental replicates of organs exposed to 5 ng/mL TGFβ1 vs. organs exposed to basal media alone. Note that the patterns generated by the two analytical methods are similar. Supplemental Figure S3. Expression of Tgfb1 in embryonic jejunum in vivo and in organ culture. QPCR measurements for Tgfb1 factored for Gapdh, expressed as the mean ± SD of three samples of freshly dissected E14 jejunum (yellow, E14–D0), freshly dissected E17 jejunum (orange, E17), and E14 rudiments cultured for 3 days in basal media alone (red, E14‐D3). Levels of Tgfβ1 transcripts fell (p = 0.01) when comparing E14 and E17 jejuna and also when comparing freshly dissected E14 organs with those cultured for 3 days [file TERM-12-252-s001.doc]

**Supplemental Results**

**Exogenous transforming growth factor-β1 enhances smooth muscle differentiation in embryonic mouse jejunal explants**

Riccardo Coletta, Neil A. Roberts, Michael J. Randles, Antonino Morabito and Adrian S. Woolf.

**Supplemental Figure S1. Venn diagrams showing the significantly up and down regulated transcripts in organ cultures administered TGFβ1 *versus* explants exposed to basal media alone.**

The number in parentheses next to each concentration of TGFβ1 is the total up- or down-regulated transcripts for that concentration. The numbers of transcripts in the Overlaps (i.e. changed in both concentration of TGFβ1 versus basal media) are also indicated.

**Supplemental Figure S2. Comparisons of changes in levels of selected transcripts measured by microarray and QPCR**.

Changes in levels of transcripts as assessed by microarray (blue) and QPCR (red) analyses. Each column is the mean (±SD) of four experimental replicates of organs exposed to 5 ng/mL TGFβ1 *versus* organs exposed to basal media alone. Note that the patterns generated by the two analytical methods are similar.

**Supplemental Figure S3. Expression of *Tgfb1* in embryonic jejunum *in vivo* and in organ culture.**

QPCR measurements for *Tgfb1* factored for *Gapdh*, expressed as the mean±SD of three samples of freshly dissected E14 jejunum (yellow, E14-D0), freshly dissected E17 jejunum (orange, E17), and E14 rudiments cultured for three days in basal media alone (red, E14-D3). Levels of *Tgfβ1* transcripts fell (P=0.01) when comparing E14 and E17 jejuna and also when comparing freshly dissected E14 organs with those cultured for three days
